# Preoperative sleep disturbance and postoperative delirium in elderly joint replacement patients: a prospective cohort study

**DOI:** 10.1186/s12893-026-03797-0

**Published:** 2026-05-07

**Authors:** Jiawei Han, Qianyu Yang, Yanan Zhao, Lu Chen, Zixuan Wang, Jiayu Zhu, Xuesen Su, Shouyuan Tian

**Affiliations:** 1 The College of Anesthesia, Shanxi Medical University, Taiyuan, Shanxi China; 2 Cancer Hospital Affiliated to Shanxi Medical University, Shanxi Medical University, Taiyuan, Shanxi China; 3 The Department of Anesthesiology, The Fifth Clinical Medical College of Shanxi Medical University, Taiyuan, Shanxi China; 4 Shanxi Key Laboratory of Geriatric Precision Anesthesia and Complication Prevention, The Fifth Clinical Medical College of Shanxi Medical University, Taiyuan, Shanxi China

**Keywords:** Postoperative delirium, Preoperative sleep disturbance, Knee replacement, Total hip replacement, Pittsburgh Sleep Quality Index

## Abstract

**Background:**

Postoperative delirium (POD) represents a frequent complication among patients undergoing joint replacement surgery. While sleep disturbance is prevalent during the perioperative period, its relationship with POD has not been fully clarified. To this end, the present study was conducted to investigate the association between preoperative sleep disturbance and POD in elderly patients undergoing joint replacement.

**Methods:**

This prospective cohort study recruited 200 patients undergoing elective knee or total hip replacement surgery between April 10th and July 15th, 2025, retrospectively registered with the Chinese Clinical Trial Registry (ChiCTR2500113120) on November 25, 2025. Specifically, preoperative subjective sleep quality was assessed using the Pittsburgh Sleep Quality Index (PSQI) on the day of admission. POD was assessed twice daily using the Chinese version of the 3-Minute diagnostic assessment for delirium based on the Confusion Assessment Method (3D-CAM) within the first postoperative week. Multivariable logistic regression was performed to examine the association between preoperative sleep disturbance and POD, adjusting for age, sex, American Society of Anesthesiologists (ASA) classification, alcohol consumption, smoking,comorbidity, education level, type of anesthesia and PCIA.

**Results:**

Preoperative sleep disturbance was observed in 69 of 200 patients (35%), with POD present in28 patients (14%). Followingmultivariable adjustment, preoperative sleep disturbance was independently associated with the occurrence of POD (*OR*: 2.84, 95% CI: 1.24–6.50, *P* = 0.013). The RCS curve revealed a significant positive linear association: as the PSQI score increased, POD risk increased gradually (*P* for overall = 0.036, *P* for nonlinear = 0.190). Exploratory subgroup analyses indicated a significant association between preoperative sleep disturbance and POD among patients under 80 years of age, females, patients with a normal BMI, and those with an ASA Ⅱ.

**Conclusion:**

In elderly patients undergoing elective knee or total hip replacement surgery, preoperative sleep disturbance is independently associated with an increased risk of POD.

**Supplementary Information:**

The online version contains supplementary material available at 10.1186/s12893-026-03797-0.

## Introduction

Postoperative delirium (POD) is an acute neurocognitive disorder arising following anesthesia and surgery. It is characterized by transient, fluctuating disturbances in attention, memory, and executive function, with onset usually occurring within the first three postoperative days [1–3]. Additionally, POD has also been reported to be associated with prolonged hospital stays and long-term cognitive impairment [1, 4]. 

Sleep represents a natural, recurrent physiological state characterized by diminished consciousness, reduced responsiveness to external stimuli, and a temporary suspension of voluntary activities. The circadian system regulates individuals’ sleep-wake cycles through the secretion of the hypnotic hormone melatonin, which peaks during the night [5]. As an essential biological process governed by circadian rhythms, sleep holds considerable significance in maintaining physiological homeostasis and preventing the development of various diseases [6, 7]. 

Sleep disturbance refers to abnormalities in sleep duration, behavioral patterns during sleep, and disruptions in sleep-wake cycles. In China, these disturbances are highly prevalent among the elderly, particularly females, involving an incidence rate of 35.9% [8]. A study has shown that over 60% of patients with mild cognitive impairment and Alzheimer’s disease(AD) suffer from at least one sleep disturbance [9]. Such disturbances encompass various dimensions of sleep, including duration, latency, and efficiency, assessable via both subjective self-reports and objective measures [10]. 

Previous studies have primarily focused on cardiac surgery or major non-cardiac surgeries. Research on the impact of preoperative sleep disturbances on POD in specific orthopedic surgeries, such as knee and hip replacements, remains largely unexplored. Elderly patients undergoing arthroplasty differ from other surgical populations in terms of baseline mobility, pain burden, postoperative immobilization, and susceptibility to delirium triggers. Accordingly, this study investigated the association between preoperative sleep disturbance and POD specifically in joint replacement patients. The findings are expected to enable clinicians to implement tailored perioperative interventions, optimize recovery outcomes, and improve postoperative rehabilitation.

## Materials and methods

### Study design and participants

This prospective cohort study was conducted at the First Hospital of Shanxi Medical University. Ethical approval was obtained from the institutional ethics committee (KYLL-2025-099), and the trial was retrospectively registered with the Chinese Clinical Trial Registry (ChiCTR2500113120) on November 25, 2025. All 200 enrolled patients (100%) completed the 7-day postoperative follow-up, with no loss to follow-up or patient withdrawal. Based on institutional data for the full calendar year 2024 (the most recent complete year prior to study initiation), the average monthly volume of elective knee or total hip replacement surgeries was 48. Revision surgeries accounted for 8.9% of all primary and revision procedures performed during this period. The overall incidence of postoperative adverse events (defined as complications occurring within 30 days post-surgery, including surgical site infection, deep vein thrombosis, prosthetic dislocation, or unplanned readmission) was 4.5%. A total of 12 surgeons performed these procedures in 2024, 6 of whom were attending surgeons.

Eligibility was assessed in patients aged 65 years or older undergoing elective knee or total hip arthroplasty from April to July 2025. All patients, or their legal representatives, provided written informed consent. Exclusion criteria included: (1) history of delirium, cognitive dysfunction, or psychosis. Cognitive function was systematically screened using the Mini-Mental State Examination (MMSE); patients with an MMSE score < 24 were considered to have cognitive dysfunction and were excluded; (2) ASA physical status classification >Ⅲ; (3) severe respiratory or cardiovascular complications, defined as: cardiovascular: New York Heart Association (NYHA) functional class III or IV, or a history of myocardial infarction within the past 6 months; respiratory: chronic obstructive pulmonary disease (COPD) with preoperative oxygen saturation < 90% on room air, or requirement for home oxygen therapy; or (4) inability to complete assessments due to severe visual or hearing impairment, language barriers, or other reasons.

### Anesthesia

Either general anesthesia (GA) or combined spinal-epidural anesthesia (CSEA) was performed based on surgical requirements, patient preference, and physical status. Anesthesia type distribution was balanced between the sleep disturbance and no sleep disturbance group to ensure comparability. GA was induced with sufentanil (0.4 µg/kg), etomidate (0.3 mg/kg), and cisatracurium (0.15 mg/kg). Upon tracheal intubation, mechanical ventilation was maintained to keep End-tidal carbon dioxide partial pressure (P_ET_CO2) between 35 and 45 mmHg. Maintenance was achieved via continuous infusion of propofol (4–6 mg/kg/h) and remifentanil (0.2–0.3 µg/kg/min), titrated to maintain a Bispectral Index (BIS) value between 40 and 60. For patients receiving CSEA, the puncture was performed at the L2-3 or L3-4 interspace. Intrathecal administration consisted of 20 mg of 0.67% hyperbaric ropivacaine. A > 20% increase in heart rate or blood pressure from baseline during surgery prompted supplementary boluses of 2% lidocaine (3–5 mL) via the epidural catheter. Following the surgery, the patient’s epidural catheter was removed. All patients undergoing combined spinal-epidural anesthesia did not receive patient controlled epidural analgesia (PCEA). The drug regimen for patient controlled intravenous analgesia (PCIA) in postoperative patients has been standardized.The regimen consisted of sufentanil 150 µg and ondansetron 16 mg diluted with 0.9% normal saline to a total volume of 100 mL (sufentanil concentration: 1.5 µg/mL). The PCIA pump was set with a background infusion of 2 mL/h, a patient-controlled bolus of 2 mL, a lockout interval of 15 min, and an hourly maximum limit of 10 mL. Apart from standardized PCIA, no additional sedatives or hypnotics were administered postoperatively to avoid confounding effects on sleep and delirium.

### Sleep disturbance assessment

Patients were instructed to complete the Chinese version of the Pittsburgh Sleep Quality Index (PSQI) questionnaires via a self-administered approach on the day of admission. PSQI comprises 18 self-rated items, categorized into seven components, namely subjective sleep quality, sleep latency, sleep duration, habitual sleep efficiency, sleep disturbances, use of sleeping medication, and daytime dysfunction. Each component is scored from 0 to 3, yielding a global score ranging from 0 to 21. Herein, the PSQI demonstrated satisfactory internal consistency, with a Cronbach’s alpha coefficient of 0.73. A cutoff score of 5, determined empirically, was used to differentiate between good and poor sleep quality [11]. Patients were divided into two groups based on the PSQI score. The sleep disturbance group was defined as a score of > 5, and the non-sleep disturbance group was defined as a score of ≤ 5 [11], with a higher score indicating more serious sleep disturbance [11]. The PSQI total score was hereby treated as a continuous variable for correlation and mediation analyses, and as a categorical variable (score ≤ 5 vs. > 5) for descriptive statistics and group comparisons.

### Postoperative delirium assessment

POD was assessed using the Chinese version of the 3-Minute diagnostic assessment for delirium using the Confusion Assessment Method (3D-CAM). This very approach is primarily used in medical settings such as emergency departments and for delirium assessments in elderly patients [12]. The evaluation consists of four aspects: (1) acute changes or repeated fluctuations in mental state; (2) attention deficit disorder; (3) changes in consciousness level; and (4) disordered thinking. Delirium was diagnosed when criteria (1), (2) and (3) or (4) were met [13]. The sensitivity and specificity of the Chinese version of 3D-CAM in evaluating delirium in elderly patients were 94.73% and 97.92% respectively [12]. To ensure assessment rigor, all investigators underwent standardized training and were qualified via a formal competency evaluation prior to the study. Inter-rater reliability was assessed between two primary investigators using a subset of 30 patients, yielding a Cohen’s kappa coefficient of 0.85. Additionally, the first assessment was conducted at 24 h post-surgery. From the 2nd to 7th day post-surgery, patients were evaluated twice daily (8:00–10:00 and 18:00–20:00) [13]. The delirium assessment investigators were blinded to the PSQI score. Furthermore, a standardized clinical pathway was implemented. The protocol specified that: (1) daily cognitive assessment be performed by trained researchers using 3D-CAM; (2) routine blood tests (including electrolytes, blood gases, and complete blood count) be performed daily within the first 48–72 h post-surgery to rule out metabolic contributors; and (3) the standardized PCIA protocol be applied to mitigate potential drug-related confounding. Patients with acute neurological events (e.g., stroke) or severe, refractory metabolic disorders during the observation period were excluded from the final analysis.

### Clinical data collection

Demographic and clinical data were systematically collected from the Electronic Medical Record (EMR) system. Demographic variables included age, sex, Body Mass Index (BMI), and educational level. Lifestyle factors were strictly defined as smoking status was categorized as current (≥ 1 cigarette/day for > 6 months), former (quit > 6 months), or never-smoker; alcohol consumption was categorized as excessive (> 14 standard drinks/week for men, > 7 for women) or none/moderate. Preoperative assessments included the PSQI score, Visual Analogue Scale (VAS) score for pain, and comorbidities (hypertension, diabetes, dyslipidemia, pulmonary diseases, coronary heart disease, and stroke). Intraoperative data comprised the anesthesia and operation duration, ASA classification, anesthesia type, infusion volume, and blood loss. To evaluate the pharmacological impact on POD, cumulative perioperative opioid dosages were recorded and converted into Morphine Milligram Equivalents (MME) using standardized conversion factors (e.g., 1 µg of sufentanil = 1 mg of MME). Furthermore, in this study, the standardized anesthetic protocol strictly avoided the use of benzodiazepines. Anticholinergic medications (e.g., atropine) were reserved solely as rescue boluses for symptomatic bradycardia and were not used as routine premedication. Additionally, postoperative data included the duration of hospitalization, the use of Patient-Controlled Intravenous Analgesia (PCIA), and POD occurrence. Postoperative complications were monitored until discharge and graded usingthe Clavien-Dindo classification to adjust for surgical morbidity in the final analysis.

### Sample size calculation

POD represents a severe and costly complication that predominantly affects individuals aged 65 years or older, with an incidence nearing 35% in orthopedic surgery populations [14]. Herein, the required sample size was calculated to assume a 35% incidence rate. The initial sample size was hereby determined to be 172 using the sample size calculation formula for observational studies (n=(6EPV*10)/35%), with 6 multivariable factors requiring 10 events per variable. Upon accounting for a 10% dropout rate, the final required sample size was 200.Additionally, a power test was conducted using G-power software, with Power (1-β) = 0.8034.

### Statistical analysis

The normality of the variable’s distribution was assessed using the Kolmogorov-Smirnov test. Continuous variables with a normal distribution were expressed as mean ± standard deviation (SD), while those with a non-normal distribution were reported as the median, interquartile range (IQR) and range. Categorical variables were presented as frequencies and percentages. Student’s *t* test and the Mann-Whitney *U* test were utilized for continuous variables, whereas the Pearson chi-square test and Fisher’s exact test were employed for categorical variables. The relationship between preoperative sleep disturbances and POD was analyzed using multivariate logistic regression analysis. A *P-*value < 0.05 was considered statistically significant in all analyses. All statistical analyses were conducted using SPSS Statistics 25.0 (IBM Corp, Armonk, NY, USA).

A restricted cubic spline (RCS) regression with the multivariate adjustments was utilized to determine the presence of a nonlinear relationship between preoperative PSQI score and POD. Concurrently, to investigate potential effect modification by age, sex, overweight, ASA classification, type of anesthesia, and PCIA on the association between sleep disturbances and POD, multiplicative interaction terms (sleep disturbance × age, sleep disturbance × sex, sleep disturbance × overweight, sleep disturbance × ASA classification, sleep disturbance × type of anesthesia, and sleep disturbance × PCIA) were incorporated into the multivariable logistic regression models for formal interaction testing.

## Results

### Characteristics of study participants

**Fig. 1:**
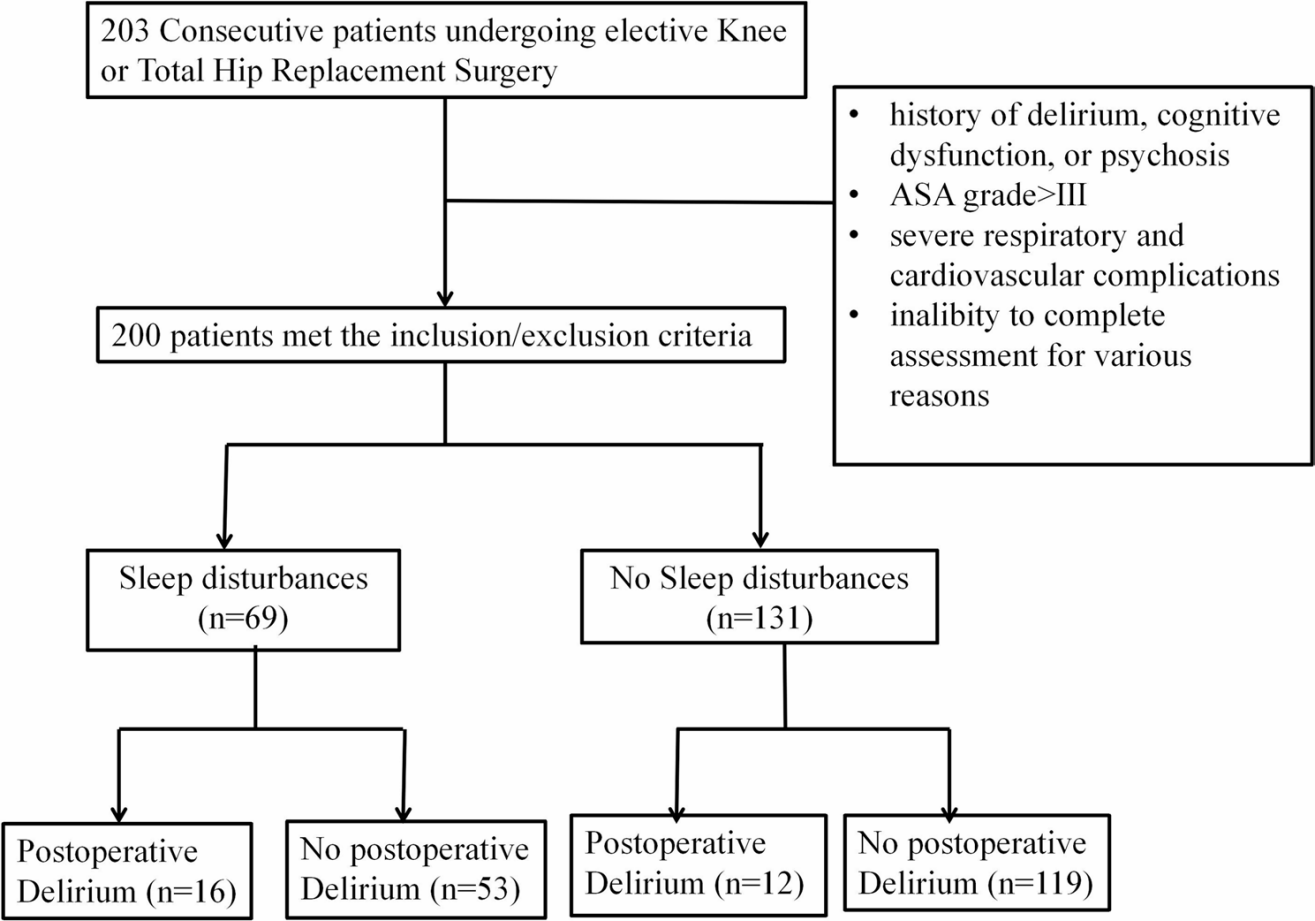
Flow chart of included population

A total of 203 patients were screened, and 200 were ultimately enrolled. Their ages ranged from 65 to 74 years, with a median age of 69 years. Among them, 83 (41.5%) were male (Fig. 1).Meanwhile, 69 (34.50%) were diagnosed with preoperative sleep disturbance based on the PSQI, and 23.19% (16/69) were diagnosed with POD. This rate was considerably higher compared to those without preoperative sleep disturbance (χ^2^ = 7.39, *P* = 0.007) (Table 1).

**Table 1:** Univariate analysis according to sleep disturbances

	All patients (*n* = 200)	No sleep disturbance (*n* = 131)	Sleep disturbance (*n* = 69)	Statistics	*P* value
Age, years	69.00 (65.00, 74.00)	68.00 (65.00, 74.00)	71.00 (65.00, 77.00)	-1.22	0.221
Body mass index, kg/m^2^	24.55 ± 3.53	24.53 ± 3.50	24.59 ± 3.62	-0.10	0.923^$^
PSQI score	4.00 (2.00, 6.00)	3.00 (2.00, 4.00)	7.00 (6.00, 9.00)	-11.68	< 0.001
VAS score	6.00 (0.00, 8.00)	6.00 (0.00, 7.00)	6.00 (0.00, 8.00)	-0.22	0.828
Male, *n* (%)	83 (41.50)	54 (41.22)	29 (42.03)	0.01	0.912*
Alcohol consumption, *n* (%)	44 (22.00)	30 (22.90)	14 (20.29)	0.18	0.672*
Smoking, *n* (%)	32 (16.00)	21 (16.03)	11 (15.94)	0.00	0.987*
Hypertension, *n* (%)	74 (37.00)	47 (35.88)	27 (39.13)	0.21	0.651*
Diabetes, *n* (%)	37 (18.50)	22 (16.79)	15 (21.74)	0.73	0.392*
Dyslipidemia, *n* (%)	15 (7.50)	9 (6.87)	6 (8.70)	0.22	0.641*
Pulmonary diseases, *n* (%)	10 (5.00)	5 (3.82)	5 (7.25)	0.51	0.474*
Coronary heart disease, *n* (%)	22 (11.00)	15 (11.45)	7 (10.14)	0.08	0.779*
Stroke, *n* (%)	10 (5.00)	7 (5.34)	3 (4.35)	0.00	1.000*
Education level, *n* (%)				-	0.706^+^
Junior middle school	94 (47.00)	59 (45.04)	35 (50.72)		
Senior high school	105 (52.50)	71 (54.20)	34 (49.28)		
Undergraduate	1 (0.50)	1 (0.76)	0 (0.00)		
Type of anesthesia, *n* (%)				0.23	0.632*
Intraspinal anesthesia	51 (25.50)	32 (24.43)	19 (27.54)		
General anesthesia	149 (74.50)	99 (75.57)	50 (72.46)		
Operation type, *n* (%)				0.03	0.855*
Knee replacement	135 (67.50)	89 (67.94)	46 (66.67)		
Total hip replacement	65 (32.50)	42 (32.06)	23 (33.33)		
Postoperative delirium, *n* (%)	28(14.00)	12(9.16)	16(23.19)	7.39	0.007*

Data are presented as *n* (%), mean ± standard deviation, or median (Interquartile range). These analyses were performed using *Chi-square test ^#^Mann-Whitney U-test, ^$^t test and ^+^Fisher’s exact tests

### Preoperative sleep disturbances and postoperative delirium

In the univariate logistic regression analysis, preoperative sleep disturbance was identified as the sole factor significantly associated with POD (*OR*: 2.99, 95% CI: 1.32–6.77, *P* = 0.020), whereas none of the other examined variables showed a statistically significant association. These factors included age, sex, alcohol consumption, smoking, hypertension, dyslipidemia, pulmonary diseases, coronary heart disease, stroke, education level, ASA classification, type of anesthesia, MME, VAS, and PCIA. In contrast, preoperative pain intensity, as measured by the VAS, exhibited no statistically significant association with POD (*OR* = 1.02, 95% CI: 0.91–1.15, *P* = 0.719) (Table 2). Based on clinical significance and previous literature, age and ASA classification are well-established predictors of postoperative delirium. Therefore, a parsimonious model adjusting for age and ASA classification was used for multivariable logistic regression analysis. In this model, preoperative sleep disturbance remained significantly associated with postoperative delirium (POD) (*OR* = 2.84, 95% CI: 1.24–6.50, *P* = 0.013). Neither age nor ASA classification was independently associated with POD in this model (both *P* > 0.05) (Table 2). With 28 POD events and 3 covariates included in the multivariable model, the events per variable (EPV) was 9.3 (28/3), slightly lower than the recommended threshold of 10. Notably, collinearity diagnostics confirmed that all variance inflation factors (VIF) were below 5, indicating no substantial multicollinearity among the independent variables.

**Table 2:** Variables associated with postoperative delirium on logistic regression analysis

Variables	Model 1		Model 2	
	*P*	OR (95%CI)	*P*	OR (95%CI)
Sleep disturbance				
No		1.00(Reference)		1.00(Reference)
Yes	0.008	2.99 (1.32 ~ 6.77)	0.013	2.84 (1.24 ~ 6.50)

### Comparison between POD and non-POD patients

Among the 200 patients enrolled, 14% (28/200) were diagnosed with POD (Table 3). Furthermore, the POD group exhibited poorer overall sleep quality, as measured by mean total PSQI score, compared to the non-POD group (*Z* = -2.41, *P* = 0.016). As summarized in Table 3, sleep problems were more prevalent among participants who developed POD compared to their counterparts who did not. The POD group had worse subjective sleep quality (*Z* = -2.34, *P* = 0.019) and shorter sleep duration ( *Z* = -2.36, *P* = 0.018) compared to the non-POD group. Additionally, sleep disturbances were more prevalent in the POD group (16 [57.14%] vs. 53 [30.81%], *P* = 0.007). Postoperative analgesic use was compared between the two groups. In the non- POD group, 68.60% (118/172) of patients utilized the standardized PCIA regimen, while in the POD group, 60.71% (17/28) used PCIA. Moreover, statistical analysis revealed no significant difference in the proportion of PCIA usage between the groups (*P* = 0.408), suggesting that the analgesic modality did not act as a confounding variable for POD development. Lastly, patients with POD experienced longer hospital stays(*P* < 0.001) compared to those without (Table 4).

**Table 3:** Baseline characteristics associated with postoperative delirium

	All patients (*n* = 200)	No postoperative delirium (*n* = 172)	Postoperative delirium (*n* = 28)	Statistics	*P* value
Age, years	69.00 (65.00, 74.00)	69.00 (65.00, 74.00)	71.00 (66.50, 79.25)	-1.72	0.085
Body mass index, kg/m^2^	24.55 ± 3.53	24.53 ± 3.52	24.71 ± 3.69	-0.26	0.796
PSQI score	4.00 (2.00, 6.00)	4.00 (2.00, 6.00)	6.00 (3.00, 8.00)	-2.41	0.016
Sleep quality score	1.00 (0.00, 1.00)	1.00 (0.00, 1.00)	1.00 (0.00, 2.00)	-2.34	0.019
Sleep latency score	1.00 (0.00, 1.00)	1.00 (0.00, 1.00)	1.00 (0.75, 2.00)	-1.53	0.127
Hours of sleep score	1.00 (0.00, 1.00)	1.00 (0.00, 1.00)	1.00 (0.75, 2.00)	-2.36	0.018
Sleep efficiency score	1.00 (0.00, 1.00)	0.00 (0.00, 1.00)	1.00 (0.00, 2.00)	-1.62	0.106
Sleep disorder score	1.00 (0.00, 1.00)	1.00 (0.00, 1.00)	1.00 (0.00, 1.00)	-0.09	0.928
Hypnotics score	0.00 (0.00, 0.00)	0.00 (0.00, 0.00)	0.00 (0.00, 0.00)	-0.46	0.644^#^
Daytime dysfunction score	0.00 (0.00, 0.00)	0.00 (0.00, 0.00)	0.00 (0.00, 1.00)	-1.92	0.055^#^
Male, *n* (%)	83 (41.50)	74 (43.02)	9 (32.14)	1.17	0.279*
Alcohol consumption, *n* (%)	44 (22.00)	38 (22.09)	6 (21.43)	0.01	0.937*
Smoking, *n* (%)	32 (16.08)	29 (16.86)	3 (10.71)	0.31	0.578*
Hypertension, *n* (%)	74 (37.00)	63 (36.63)	11 (39.29)	0.07	0.787*
Diabetes, *n* (%)	37 (18.50)	29 (16.86)	8 (28.57)	2.19	0.139*
Dyslipidemia, *n* (%)	15 (7.50)	11 (6.40)	4 (14.29)	1.17	0.279*
Pulmonary diseases, *n* (%)	10 (5.00)	7 (4.07)	3 (10.71)	1.06	0.304*
Coronary heart disease, *n* (%)	22 (11.00)	18 (10.47)	4 (14.29)	0.07	0.784*
Stroke, *n* (%)	10 (5.00)	9 (5.23)	1 (3.57)	0.00	1.000*
Education level, *n* (%)				-	0.730^+^
Junior middle school	94 (47.00)	82 (47.67)	12 (42.86)		
Senior high school	105 (52.50)	89 (51.74)	16 (57.14)		
Undergraduate	1 (0.50)	1 (0.58)	0 (0.00)		
Sleep disturbance, *n* (%)	69 (34.50)	53 (30.81)	16 (57.14)	7.39	0.007*
VAS, M (Q₁, Q₃)	6.00 (0.00, 8.00)	6.00 (0.00, 7.25)	6.00 (0.00, 8.00)	-0.35	0.725^#^

Data are presented as *n* (%), mean ± standard deviation, or median (Interquartile range). These analyses were performed using *Chi-square test, ^#^Mann-Whitney U-test, ^$^t test -test and ^+^Fisher’s exact tests

**Table 4:** Surgical characteristics associated with postoperative delirium

	All Patients (*n* = 200)	No postoperative delirium (*n* = 172)	Postoperative delirium (*n* = 28)	Statistics	*P* value
Duration of surgery, minutes	108.00 (86.00, 132.00)	107.50 (86.00, 133.50)	110.00 (86.50, 126.75)	-0.13	0.895^#^
Duration of anesthesia, minutes	144.50 (117.50, 166.50)	144.50 (118.00, 168.00)	144.00 (113.75, 165.00)	-0.17	0.864^#^
Intraoperative infusion volume, mL	750.00 (500.00, 1000)	750.00 (500.00, 1000.00)	750.00 (500.00, 1000.00)	-0.47	0.637^#^
Blood loss during surgery, mL	100.00 (100.00, 150.00)	100.00 (100.00, 150.00)	100.00 (80.00, 150.00)	-0.96	0.336^#^
Duration of hospitalization, days	6.00 (5.00, 6.00)	6.00 (5.00, 6.00)	8.00 (7.00, 8.25)	-8.00	< 0.001^#^
PCIA, *n* (%)	135 (67.50)	118 (68.60)	17 (60.71)	0.68	0.408*
ASA classification, *n* (%)				0.86	0.355*
Ⅱ	109 (54.50)	96 (55.81)	13 (46.43)		
Ⅲ	91 (45.50)	76 (44.19)	15 (53.57)		
Type of anesthesia, *n* (%)				0.76	0.385*
Intraspinal anesthesia	51 (25.50)	42 (24.42)	9 (32.14)		
General anesthesia	149 (74.50)	130 (75.58)	19 (67.86)		
Operation type, *n* (%)				0.00	0.965*
Knee replacement	135 (67.50)	116 (67.44)	19 (67.86)		
Total hip replacement	65 (32.50)	56 (32.56)	9 (32.14)		
MME, mg	150.00 (25.00, 170.00)	150.00 (25.00, 170.00)	150.00 (20.00, 170.00)	-1.09	0.275^#^

*Abbreviations*: *PCIA* Patient Controlled Intravenous Analgesia, *MME* Morphine Milligram Equivalent

These analyses were performed using *Chi-square test, and ^#^Mann-Whitney U-test

### Restricted cubic splines analysis

**Fig. 2:**
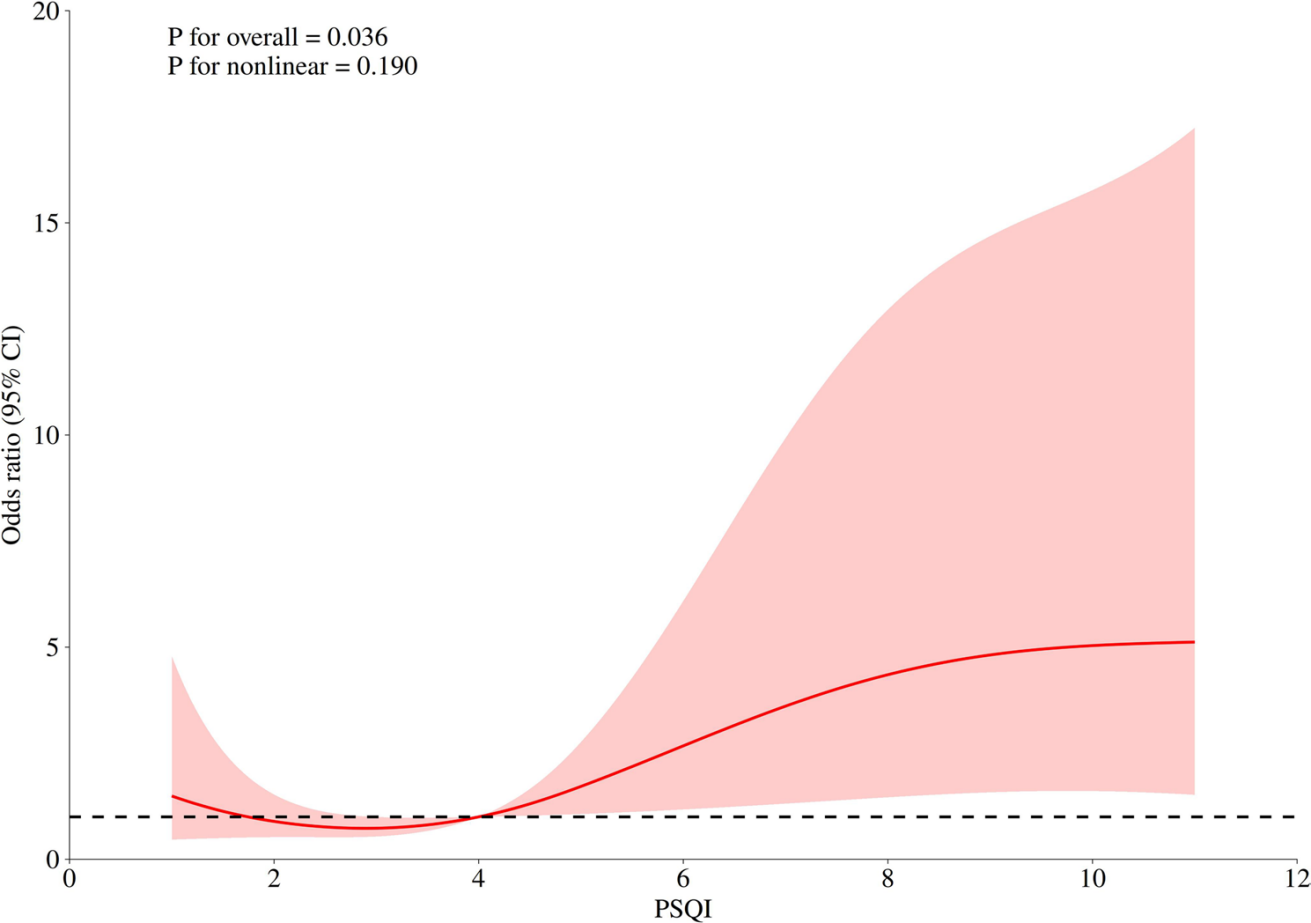
Restricted cubic spline (RCS) curve between PSQI score and POD. Abbreviations: OR: 2.86, 95% CI: 1.18 ~ 6.91

A restricted cubic spline (RCS) model with knots positioned at the 5th, 35th, 65th, and 95th percentiles of PSQI score was employed to examine the association between preoperative PSQI score and the risk of POD in elderly patients undergoing elective knee or total hip replacement. Upon adjusting for multiple potential confounders including age, sex, alcohol consumption, smoking, hypertension, dyslipidemia, pulmonary diseases, coronary heart disease, stroke, education level, ASA classification, type of anesthesia and PCIA, the RCS curve uncovered a significant positive linear association. Specifically, using a PSQI score of 5 (the clinical threshold for poor sleep) as the reference, the risk of POD gradually increased with higher PSQI scores (*P* for overall = 0.036, *P* for nonlinear = 0.190) (Fig. 2).

### Subgroup analysis of the association between preoperative sleep disturbances and POD

**Fig. 3:**
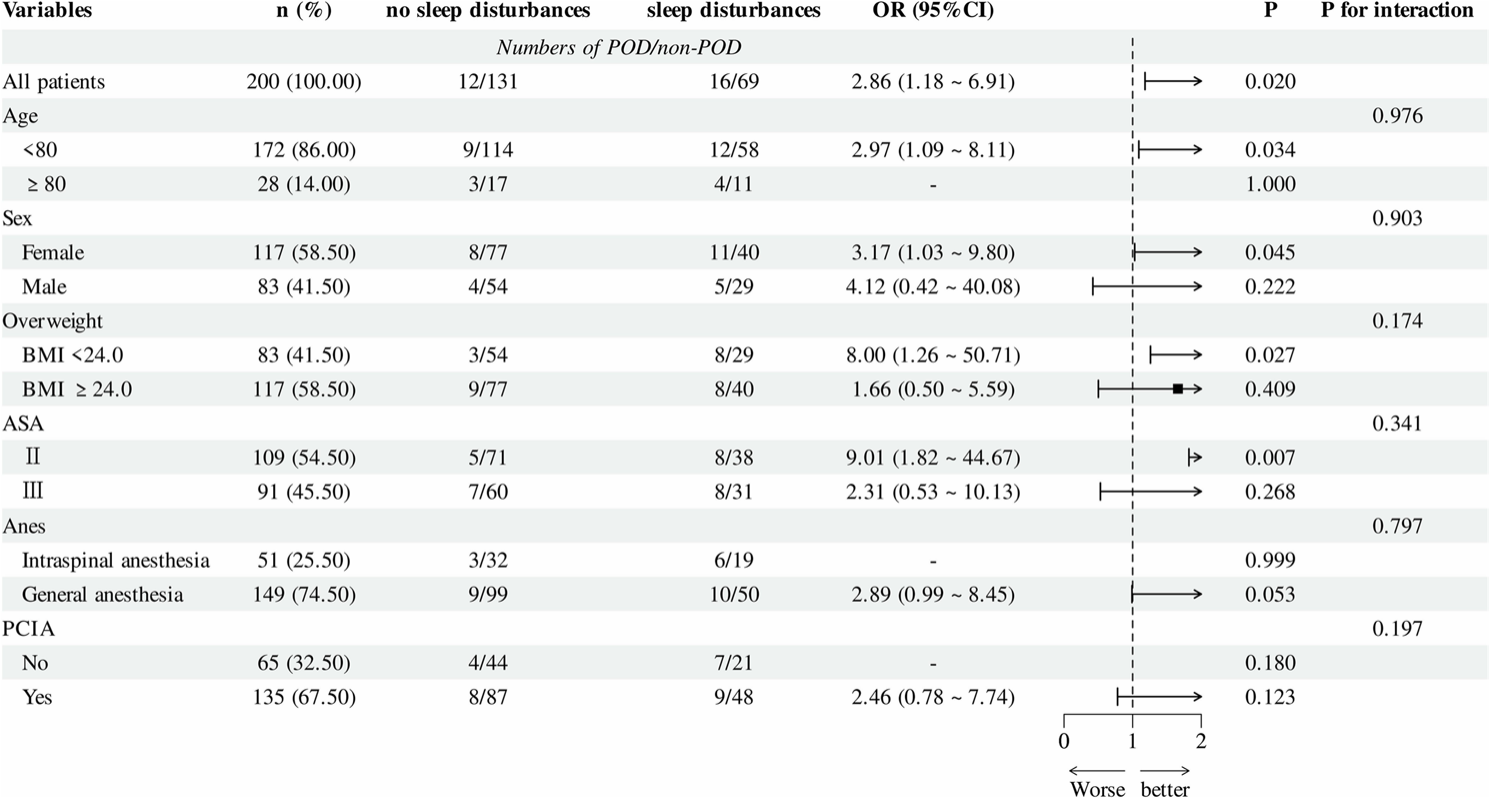
Forest plots of *OR*s by patient subgroups. Abbreviations: PCIA, Patient Controlled Intravenous Analgesia. Anes, Type of anesthesia

To assess potential effect modifiers, subgroup analyses were hereby conducted based on age, sex, overweight status, ASA, anesthesia type, and PCIA. A significant association between preoperative sleep disturbance and POD was observed only in the following subgroups: individuals under 80 years of age, females, patients with a normal BMI, and those with an ASA classification of Ⅱ. However, no statistically significant interaction was detected for any of the subgroups (all *P* for interaction > 0.05). This indicates that between-group differences may be attributable to chance or sample size imbalance, with no evidence supporting these variables as effect modifiers (Fig. 3).

## Discussion

This study established a significant association between preoperative sleep disturbance and POD in elderly patients undergoing elective knee or total hip replacement surgery. Results confirmed that patients with preoperative sleep disturbance (PSQI > 5) were at an increased risk of developing POD. A recent study by Yücel et al. highlighted the predictive value of preoperative functional assessments, including the 30-second sit-to-stand test and the timed up-and-go test, for postoperative outcomes [15]. Our findings further support the importance of comprehensive preoperative evaluation—incorporating sleep assessment—for risk stratification and perioperative management.

While preoperative sleep disturbance emerged as the sole statistically significant predictor of POD in the present multivariable logistic regression analysis, several established risk factors—including age, ASA classification, and comorbidities—did not exhibit statistical significance. This may be attributable to limited statistical power stemming from the sample size, or to reduced variability within a relatively homogeneous elective surgical cohort. Alternatively, it sleep disturbance may potentially mediate part of the risk typically attributed to these traditional factors. Importantly, the absence of statistical significance did not denote clinical irrelevance; age and comorbidity burden were still recognized as essential components of perioperative risk assessment.

In the present study, the prevalence of preoperative sleep disturbances was 35%, a figure aligning closely with epidemiological benchmarks for the geriatric population. Comprehensive meta-analyses have reported that approximately 35.9% of community-dwelling older adults in China [16] and 30% to 48% globally experience similar sleep issues [17]. Herein, POD occurred in 23% of patients with preoperative sleep disturbances. To our knowledge, this is the first study investigating the correlation between preoperative sleep disturbances and POD in elderly patients undergoing elective knee or total hip replacement surgery. The observed POD incidence in this study is consistent with results in prior research, which has reported rates ranging from 21.7% to 50% [18, 19]. Furthermore, a meta-analysis of 27 observational studies demonstrated that older adults with self-reported sleep disorders exhibited a 1.68-fold higher risk of neurocognitive impairment [20]. Bubu et al.’s recent meta-analysis similarly documented a 3.78-fold greater risk of preclinical Alzheimer’s disease in individuals with sleep disturbances (95% CI: 2.27–6.30) [20]. 

In this study, preoperative sleep disturbance was independently associated with an increased risk of POD. While the precise mechanisms linking sleep disturbance to POD could not be determined by the present clinical data, several pathways have been proposed in the literature that may help explain this association. Emerging evidence suggests that sleep disturbances may promote the inflammatory response associated with amyloid-beta (Aβ) deposition [21, 22], a pathway proposed as a biological mechanism linking sleep disruption to an increased risk of mild cognitive impairment (MCI) in the preclinical stage of AD [23, 24]. Furthermore, Aβ plaque deposition may also disrupt the sleep-wake cycle [25], suggesting a vicious cycle and bidirectional relationship between sleep and cognition. It can be accordingly inferred that the physiological mechanisms underlying preoperative sleep disturbance contribute to developing POD. Given that this study did not assess biomarkers of neuroinflammation or amyloid pathology, their role in POD pathogenesis remains speculative.

While subgroup analysis revealed no significant interaction between preoperative sleep disturbance and POD across all subgroups, the association was comparatively stronger among female patients. This suggests the presence of gender-related physiological differences in susceptibility to POD. A prospective study by Oh et al. demonstrated that among patients with acute hip fractures, the incidence of POD was significantly higher in males than in females (44.8% vs. 30.2%, *P* = 0.004). Furthermore, male sex remained a significant independent predictor of POD even following adjustment for other preoperative risk factors [26]. Given these findings, the relationship between preoperative sleep disturbance and POD may vary across sexes. Additionally, a significant association between preoperative sleep disturbance and POD was observed exclusively in patients with a lower ASA grade, suggesting that these patients may be more vulnerable to the adverse effects of preoperative sleep disturbances. A prospective observational study by Maria showed that 72% of patients having developed delirium had preoperative ASA grades ≥ 3, with 13% classified as grade 4 [27]. Based on this, an ASA grade of 3 or higher is considered a risk marker for POD [28]. The ASA physical status classification has been incorporated into the *European Society of Anesthesiology and Intensive Care* (ESAIC) guidelines for POD as a potential indicator of comorbid burden [29]. Increased ASA grade corresponds to a higher comorbidity burden and diminished functional reserve. Therefore, the impact of sleep disturbances on POD may be less noticeable or overshadowed by these more severe underlying conditions. In older patients, the burden of older age may exceed the adverse effects of preoperative sleep disturbances on POD. These findings underscore the importance of accounting for individual factors when assessing POD risk and managing those with combined preoperative sleep disturbances.

While opioids such as sufentanil are known to potentially alter sleep architecture, a standardized low-concentration PCIA regimen was hereby utilized to minimize such effects. The comparable usage rates between the POD and non-POD groups further indicated that the pharmacological intervention was not the primary driver of delirium or sleep-related adverse events in the present cohort.

In this study, preoperative sleep disturbance was identified as an independent risk factor for POD, which carried direct clinical implications. Patients with poor preoperative sleep faced a significantly higher risk of developing delirium, underscoring the potential of sleep optimization as a modifiable target for prevention. This is particularly relevant, as previous research has estimated that 30% to 40% of delirium cases may be preventable through targeted strategies [30]. The risk factor identified in this study aligns with this evidence base and suggests a specific, actionable approach: preoperative sleep optimization. Clinical care strategies for elderly postoperative patients, along with the perioperative use of melatonin, have been demonstrated to reduce the incidence of POD [31, 32]. If future trials confirm that such approaches effectively reduce POD incidence, the clinical impact could be substantial, including shorter hospital stays, reduced healthcare costs, and improved postoperative recovery. These outcomes align with the broader goal of delirium prevention highlighted previously.

However, this study has several limitations that merit acknowledgment. First, this study only focused on the sleep assessment of patients upon admission, neglecting an in-depth analysis of perioperative sleep patterns. Second, the PSQI and 3D-CAM are self-report questionnaires for sleep quality and delirium, possibly inducing some biases in POD diagnosis. While the PSQI was designed to assess sleep quality over the previous month, questionnaires were administered at hospital admission. As a result, the assessment window captured the immediate preoperative period, during which sleep quality may have been adversely affected by transient factors, notably preoperative anxiety and disease-related pain. Data on postoperative sleep quality were not proactively collected, limiting the ability to distinguish whether postoperative delirium was directly triggered by pre-existing chronic sleep disturbance or exacerbated by acute postoperative sleep fragmentation. Hence, future studies incorporating objective, longitudinal sleep monitoring, such as with polysomnography, should be carried out to further elucidate the complex interplay between preoperative and postoperative sleep patterns postoperative delirium pathogenesis. Third, a prospective design with pre-specified primary outcomes and a predefined protocol was hereby employed, yet the retrospective registration was still a methodological limitation, introducing reporting biases. Fourth, by excluding high-risk patients failing to complete the assessment, such as those with severe cognitive impairment or a history of psychiatric disorders, this study may have introduced selection biases. Consequently, the overall incidence of postoperative delirium could be underestimated. Additionally, autonomic dysfunction, increasingly prevalent with age, is a recognized factor in postoperative delirium pathogenesis. It can compromise cerebral autoregulation, heighten neuroinflammatory responses, and reduce the brain’s resilience to surgical stress, thereby influencing delirium risk. Consequently, future prospective studies should incorporate standardized measures of autonomic function, such as heart rate variability or baroreflex sensitivity. This could not only clarify its role in POD but also help identify patients who may benefit from targeted perioperative interventions. Finally, the single-center design and inclusion of only a Chinese cohort may restrict the generalizability of the present findings. Given that patient characteristics, clinical practices, and genetic backgrounds may differ across populations and healthcare systems, the present results should be extrapolated to non-Chinese populations with caution, thereby warranting validation in multicenter, multi-ethnic studies.

## Conclusions

The present findings suggest that preoperative sleep disturbances may be independently associated with the development of POD in elderly patients undergoing elective knee or total hip replacement surgery.

## Supplementary Information

Supplementary Material 1.

## Data Availability

The datasets used and analyzed during the current study are available from the corresponding author upon reasonable request.
