# Supplementary material for: Preoperative sleep disturbance and postoperative delirium in elderly joint replacement patients: a prospective cohort study
Source: BMC Surg. 2026 May 7;26:441. doi: 10.1186/s12893-026-03797-0 (PMC13321631; doi:10.1186/s12893-026-03797-0)
Supplement: Supplementary file 1 — Supplementary Material 1. [file 12893_2026_3797_MOESM1_ESM.docx]

Supplemental Table 1 Variables Associated with Postoperative Delirium on Univariate Logistic Regression Analysis

| Variables | β | S.E | Z | *P* | *OR* (95%CI) |
| --- | --- | --- | --- | --- | --- |
|  |  |  |  |  |  |
| BMI | 0.02 | 0.06 | 0.26 | 0.795 | 1.02 (0.91 ~ 1.14) |
| Age | 0.05 | 0.03 | 1.93 | 0.053 | 1.05 (1.00 ~ 1.11) |
| Sex |  |  |  |  |  |
| Female |  |  |  |  | 1.00 (Reference) |
| Male | -0.47 | 0.43 | -1.08 | 0.281 | 0.63 (0.27 ~ 1.47) |
| Alcohol consumption |  |  |  |  |  |
| No |  |  |  |  | 1.00 (Reference) |
| Yes | -0.04 | 0.50 | -0.08 | 0.937 | 0.96 (0.36 ~ 2.54) |
| Smoking |  |  |  |  |  |
| No |  |  |  |  | 1.00 (Reference) |
| Yes | -0.52 | 0.64 | -0.81 | 0.415 | 0.59 (0.17 ~ 2.09) |
| Hypertension |  |  |  |  |  |
| No |  |  |  |  | 1.00 (Reference) |
| Yes | 0.11 | 0.42 | 0.27 | 0.787 | 1.12 (0.49 ~ 2.54) |
| Diabetes |  |  |  |  |  |
| No |  |  |  |  | 1.00 (Reference) |
| Yes | 0.68 | 0.47 | 1.46 | 0.144 | 1.97 (0.79 ~ 4.91) |
| Dyslipidemia |  |  |  |  |  |
| No |  |  |  |  | 1.00 (Reference) |
| Yes | 0.89 | 0.62 | 1.43 | 0.153 | 2.44 (0.72 ~ 8.28) |
| Pulmonary diseases |  |  |  |  |  |
| No |  |  |  |  | 1.00 (Reference) |
| Yes | 1.04 | 0.72 | 1.44 | 0.150 | 2.83 (0.69 ~ 11.66) |
| Coronary heart disease |  |  |  |  |  |
| No |  |  |  |  | 1.00 (Reference) |
| Yes | 0.35 | 0.59 | 0.60 | 0.551 | 1.43 (0.44 ~ 4.57) |
| Stroke |  |  |  |  |  |
| No |  |  |  |  | 1.00 (Reference) |
| Yes | -0.40 | 1.07 | -0.37 | 0.710 | 0.67 (0.08 ~ 5.51) |
| Education level |  |  |  |  |  |
| Junior high school |  |  |  |  | 1.00 (Reference) |
| Senior high school | 0.21 | 0.41 | 0.50 | 0.617 | 1.23 (0.55 ~ 2.75) |
| Undergraduate | -13.64 | 1455.40 | -0.01 | 0.993 | 0.00 (0.00 ~ Inf) |
| Sleep disturbances |  |  |  |  |  |
| No |  |  |  |  | 1.00 (Reference) |
| Yes | 1.10 | 0.42 | 2.64 | 0.008 | 2.99 (1.32 ~ 6.77) |
| ASA classification |  |  |  |  |  |
| Ⅱ |  |  |  |  | 1.00 (Reference) |
| Ⅲ | 0.38 | 0.41 | 0.92 | 0.357 | 1.46 (0.65 ~ 3.25) |
| Type of anesthesia |  |  |  |  |  |
| Intraspinal anesthesia |  |  |  |  | 1.00 (Reference) |
| General anesthesia | -0.38 | 0.44 | -0.87 | 0.386 | 0.68 (0.29 ~ 1.62) |
| PCIA |  |  |  |  |  |
| No |  |  |  |  | 1.00 (Reference) |
| Yes | -0.35 | 0.42 | -0.82 | 0.410 | 0.71 (0.31 ~ 1.61) |
| MME | -0.00 | 0.00 | -0.96 | 0.335 | 1.00 (0.99 ~ 1.00) |
| VAS | 0.02 | 0.06 | 0.36 | 0.719 | 1.02 (0.91 ~ 1.15) |

Abbreviations: PCIA, Patient Controlled Intravenous Analgesia; OR, Odds Ratio; CI: Confidence Interval；MME，Morphine Milligram Equivalents.

Supplemental Table 2 Variables Associated with Postoperative Delirium on Multivariate Logistic Regression Analysis

| Variables | β | S.E | Z | *P* | *OR* (95%CI) |
| --- | --- | --- | --- | --- | --- |
|  |  |  |  |  |  |
| Intercept | -5.27 | 2.13 | -2.47 | 0.013 | 0.01 (0.00 ~ 0.34) |
| Age | 0.04 | 0.03 | 1.33 | 0.184 | 1.04 (0.98 ~ 1.11) |
| Sleep disturbance |  |  |  |  |  |
| No |  |  |  |  | 1.00 (Reference) |
| Yes | 1.04 | 0.42 | 2.47 | 0.013 | 2.84 (1.24 ~ 6.50) |
| ASA classification |  |  |  |  |  |
| Ⅱ |  |  |  |  | 1.00 (Reference) |
| Ⅲ | 0.11 | 0.48 | 0.23 | 0.821 | 1.12 (0.43 ~ 2.87) |

Abbreviations: OR, Odds Ratio; CI: Confidence Interval.
